# Development of Sulfur-Doped Graphitic Carbon Nitride for Hydrogen Evolution under Visible-Light Irradiation

**DOI:** 10.3390/nano13010062

**Published:** 2022-12-23

**Authors:** Tamer M. Khedr, Said M. El-Sheikh, Maya Endo-Kimura, Kunlei Wang, Bunsho Ohtani, Ewa Kowalska

**Affiliations:** 1 Institute for Catalysis, Hokkaido University, N21, W10, Sapporo 001-0021, Japan; 2 Nanomaterials and Nanotechnology Department, Central Metallurgical Research and Development Institute (CMRDI), Cairo 11421, Egypt; 3 Faculty of Chemistry, Jagiellonian University, Gronostajowa 2, 30-387 Kraków, Poland

**Keywords:** S-doped g-C_3_N_4_, 2D nanostructure, porous materials, RDB-PAS, H_2_ generation

## Abstract

Developing eco-friendly strategies to produce green fuel has attracted continuous and extensive attention. In this study, a novel gas-templating method was developed to prepare 2D porous S-doped g-C_3_N_4_ photocatalyst through simultaneous pyrolysis of urea (main g-C_3_N_4_ precursor) and ammonium sulfate (sulfur source and structure promoter). Different content of ammonium sulfate was examined to find the optimal synthesis conditions and to investigate the property-governed activity. The physicochemical properties of the obtained photocatalysts were analyzed by X-ray diffraction (XRD), field emission-scanning electron microscopy (FE-SEM), scanning transmission electron microscopy (STEM), specific surface area (BET) measurement, ultraviolet-visible light diffuse reflectance spectroscopy (UV/vis DRS), X-ray photoelectron spectroscopy (XPS), photoluminescence (PL) spectroscopy and reversed double-beam photo-acoustic spectroscopy (RDB-PAS). The as-prepared S-doped g-C_3_N_4_ photocatalysts were applied for photocatalytic H_2_ evolution under vis irradiation. The condition-dependent activity was probed to achieve the best photocatalytic performance. It was demonstrated that ammonium sulfate played a crucial role to achieve concurrently 2D morphology, controlled nanostructure, and S-doping of g-C_3_N_4_ in a one-pot process. The 2D nanoporous S-doped g-C_3_N_4_ of crumpled lamellar-like structure with large specific surface area (73.8 m^2^ g^−1^) and improved electron−hole separation showed a remarkable H_2_ generation rate, which was almost one order in magnitude higher than that of pristine g-C_3_N_4_. It has been found that though all properties are crucial for the overall photocatalytic performance, efficient doping is probably a key factor for high photocatalytic activity. Moreover, the photocatalysts exhibit significant stability during recycling. Accordingly, a significant potential of S-doped g-C_3_N_4_ has been revealed for practical use under natural solar radiation.

## 1. Introduction

The combustion of fossil fuels (primary energy source globally) and respective environmental pollution thereof keep increasing with an ever-growing human population and urbanization [1]. This, together with the gradual exhaustion of nonrenewable fuel sources, has motivated the scholars to develop novel materials and systems (clean, sustainable and renewable) as an alternative to fossil fuels, to face the energy crisis while declining environmental pollution [2]. As a promising energy carrier, hydrogen (H_2_) has attracted huge attention since it is eco-friendly and has high energy density (i.e., 122 KJ g^−1^). Moreover, it could be easily stored, transported over long distances and produced from clean and abundant sources (e.g., water splitting with the help of solar or wind energy) [3]. However, the current technologies for H_2_ generation are significantly restricted by serious drawbacks, i.e., low purity, toxicity, high cost, multi-step operation and continuous energy feedings [2,4,5]. Therefore, the development of eco-friendly and applicable alternative methods is essential. For example, the photocatalytic generation of H_2_ under solar radiation (on the surface of semiconductor photocatalysts) has attracted tremendous interest as a promising strategy to address the current environmental and energy crisis, because of high activity and performance, simple operation and fast kinetics [3]. First, H_2_ generation with photocatalytic semiconductor materials was confirmed in 1972 by Fujishima and Honda [6]. Since then, plenty of semiconductors have been developed to assess their efficiency and performance for hydrogen evolution [7,8,9,10,11]. It should be pointed out that optimizing the band structure of the photocatalytic materials plays a crucial and key role in improvement of the photocatalytic efficiency by fostering charge carriers’ separation, as well as preventing photo-corrosion and providing long-term stability. Accordingly, two-dimensional (2D) architecture nanomaterials have attracted great attention as a promising candidate for highly efficient photocatalytic reactions (such as water splitting), since their layered structure could suppress the charge carriers’ recombination, magnify the specific surface area, enhance light absorption ability and improve the efficiency of charge carriers’ transfer [12,13,14,15]. Graphene was the first 2D material exhibiting the distinctive electronic, mechanical and optical properties [14,16]. Henceforth, a multitude of 2D materials (e.g., 2D carbon-based materials and 2D transition metal dichalcogenides) have been explored to evaluate their potential for various photocatalytic applications. It should be pointed out that theoretical methods (such as discrete Fourier transform (DFT)- based theoretical method) are very important for understanding the synergies between morphological and structural issues, as well as the stability, etc., and thus addresses well experimental issues and exploring potential applications [17,18]. Among 2D photocatalytic materials, graphitic carbon nitride (g-C_3_N_4_ (CN)) has outstanding photoelectrochemical characteristics, i.e., good chemical and thermal stability, and convenient energy bandgap (2.7 eV) [19,20,21]. Furthermore, it is eco-friendly, earth-abundant and easily prepared by thermal polymerization of inexpensive nitrogen-rich raw materials (such as melamine, urea, thiourea, cyanamide, dicyandiamide) [19,20,21]. Regrettably, the photocatalytic activity of the pristine CN composed of 2D stacked layers, which is commonly prepared by thermal polymerization, is still dissatisfactory because of low electrical conductivity, a modest degree of polymerization (i.e., unfavorable structural defects), large agglomeration, large grain size (i.e., small specific surface area), low pore volume, low content of active sites, rapid recombination of photoinduced electron-hole pairs, slow charge mobility and low light-absorption ability [19,20,22]. To address these limitations, several strategies such as nanostructure elaboration (e.g., 2D nanosheets and nanoporous architectures [23,24,25,26,27], doping (metal [28], non-metal [29] and self [30]), construction of g-C_3_N_4_-based heterojunctions [31,32] and so forth, have been proposed. Among them, the nanostructure architecture could increase the specific surface area, provide more active sites, improve the light-absorption ability and boost the charge carriers’ separation and transfer, therefore enhancing the photocatalytic activity [25,26,27]. Accordingly, various methods have been used to fabricate the nanostructured g-C_3_N_4_, such as exfoliation (via chemical and mechanical treatment) and hard- or soft-templating. Nevertheless, these strategies are time-consuming, including multistep procedures, involving the participation of toxic compounds, and with the removal of templates possibly resulting in unfavorable structural defects and even nanostructure collapse [25,26,33,34]. Accordingly, the new method by gas (bubble)-templating using ammonium salts (halide, carbonate and sulfate) has recently been proposed for the preparation of nanostructured g-C_3_N_4_ [25,26,33,34]. For example, Fei et al. used ammonium chloride (NH_4_Cl) as a gas (ammonia) template to prepare porous g-C_3_N_4_ nanomaterial with a large specific surface area (139.3 m^2^ g^−1^), which was approximately 14 times higher than that of bulk g-C_3_N_4_ (10.1 m^2^ g^−1^) [35]. This nanostructure resulted in complete degradation of Rhodamine B (RhB) during half an hour of vis irradiation, while only 23% RhB was decomposed on the reference sample (bulk g-C_3_N_4_). It is thought that the nanostructure engineering improves the photocatalytic activity of g-C_3_N_4_ because of the formation of large specific surface area, the introduction of many reactive sites and the improvement of the charge carriers’ separation [25,26,33,34]. However, a decrease in the size (nanostructured g-C_3_N_4_) causes an increase in the energy bandgap, hence reducing the light-absorption ability and thus confining the improvement of photocatalytic activity to some extent [25,35]. As another effective modification strategy, metal/non-metal doping could modify the electronic structure of g-C_3_N_4_, i.e., narrowing the bandgap (broadening the visible-light absorption) and creating more active sites, resulting in the enhancement of charge carriers’ separation efficiency, thus improving the overall performance [29,36,37,38]. Although the doping with metals (e.g., Fe [39], Cu [40], Na [41], K [41,42], Pt [43], Au [44]) could improve the efficiency of electron capture, it might also cause a decrease in the stability (due to active nature of metals [45]). Accordingly, non-metal doping (e.g., S, C, N, O and P) seems to be more attractive, as it increases photocatalytic activity of g-C_3_N_4_ without worsening its stability [29,37,45]. Indeed, sulfur doping has shown to be an effective strategy for tuning the electronic structure and improving the charge carriers’ separation efficiency of g-C_3_N_4_ photocatalyst, hence boosting its photocatalytic activity (Table 1) [25,46,47,48,49,50,51]. For instance, sulfur was successfully incorporated into the g-C_3_N_4_ structure using H_2_S gas at 723 K [47]. Although S-doped g-C_3_N_4_ photocatalyst displayed a remarkable photocatalytic activity towards H_2_ generation (eight times higher than that by bare g-C_3_N_4_ photocatalyst under vis irradiation), the use of H_2_S is not eco-friendly.

Based on the aforementioned issues, the development of an eco-friendly one-step method for the preparation of non-metal modified 2D porous g-C_3_N_4_ has become highly important. Here, the simple method, i.e., simultaneous pyrolysis of urea (raw precursor) and (NH_4_)_2_SO_4_ (S source and guide structure), is proposed to prepare 2D porous S-doped g-C_3_N_4_ photocatalyst. The improved photocatalytic activity of this material could be discussed considering several aspects, including: (i) formation of 2D morphology (enlarging the surface area and improving the charge carriers’ transfer and separation efficiency), (ii) formation of mesoporous structure with high specific surface area (more reactive sites) and (iii) extending the visible light-harvesting (by reducing the energy gap with sulfur doping). Therefore, it has been proposed that 2D porous S-doped g-C_3_N_4_ semiconductor would be a promising VLA photocatalyst for different environmental and energy applications.

## 2. Materials and Methods

### 2.1. Materials 

Urea (CH_4_N_2_O, 99%), ammonium sulfate (AS, (NH_4_)_2_SO_4_, 99.5%), triethanolamine (TEOA, C_6_H_15_O_3_N, 99.5%), ethanol (EtOH, 99.5%), hydrochloric acid (HCl, 35–37%), sodium hydroxide (NaOH, 95%) and chloroplatinic acid (H_2_PtCl_6_·6H_2_O, 98.5%) were obtained from Wako Pure Chemical Co., Ltd. (Osaka, Japan). All the chemicals were used without further purification. Ultrapure water (UPW) from a Direct-Q Millipore system was used in all experiments.

### 2.2. Preparation of S-Doped g-C_3_N_4_ Photocatalyst

The 2D S-doped mesoporous g-C_3_N_4_ photocatalyst was synthesized by facile gas-templating-based thermal polymerization, using a mixture of urea and AS (different contents). In a typical procedure, a mixture of urea and AS powders with different molar ratio of AS/urea (i.e., 0.1, 0.2, 0.3 and 0.4) were grinded thoroughly in an agate mortar for 30 min. Then, the mixture was placed in a 30-mL ceramic crucible with a cover and heated in the presence of air at 550 °C for 3 h with a ramp rate of 2.2 °C min^−1^ in a muffle furnace. The obtained sample was rinsed several times with UPW and EtOH and then dried at 60 °C. For comparison, the pristine g-C_3_N_4_ (PCN) photocatalyst was also prepared in the same manner, but without AS. The 2D porous S-doped g-C_3_N_4_ photocatalysts with different molar ratio of AS/urea, i.e., 0.1, 0.2, 0.3 and 0.4 were labeled as *x*SCN, where *x* corresponds to the molar ratio of AS to urea.

### 2.3. Characterization of S-Doped g-C_3_N_4_ Photocatalyst

The crystalline properties of obtained samples were investigated by X-ray powder diffraction (XRD) analysis, measured on Rigaku intelligent XRD SmartLab with a Cu target, Rigaku, LTD., Tokyo, Japan (accelerating voltage: 40 kV, emission current: 30 mA). Samples were analyzed between 10° and 90° at 1°/min scan speed and scan step of 0.008°. The crystal structures were estimated with Rigaku PDXL software (Version 2.6.1.2, Rigaku, LTD., Tokyo, Japan, 2007–2015).

The surface morphology was investigated by field emission-scanning electron microscopy, i.e., FE-SEM (carried out on JSM-7400F, JEOL, Tokyo, Japan) under a high vacuum. Images were acquired in a wide range of magnifications in secondary electron imaging mode (SEI). Moreover, scanning transmission electron microscopy (STEM; recorded on HD-2000, Hitachi, Tokyo, Japan) was also used for three different modes: secondary electron image (SE), Z contrast image (ZC) and phase contrast image (TE). The powder samples (for FE-SEM and STEM analysis) were spread on carbon paste and dried under a vacuum overnight.

The specific surface area and the pore size distribution were investigated by nitrogen adsorption-desorption isotherms at 77 K using the Brunauer-Emmett-Teller (BET) formula and Brunauer-Joyner-Hallenda (BJH) analysis, respectively (measured using Quanta Chrome Instruments, NOVA 2000 series, Anton Paar Ltd., St Albans, UK).

The photoabsorption characteristics were analyzed with ultraviolet-visible light diffuse reflectance spectroscopy (UV-vis-DRS), carried out on JASCO V-670, equipped with a PIN-757 integrating sphere, JASCO, LTD., Pfungstadt, Germany. Barium sulfate was used as a reference for UV-vis-DRS analysis. The diffuse reflectance mode (R) was converted into the Kubelka-Munk function F(R) to separate the extent of light absorption from scattering. The band gap values were determined by plotting the (F(R) E)^1/2^) versus the energy (E) of the exciting light, where F(R) × E1/2 = ((1 − R)2/2R × E)1/2 [52].

The surface properties and the oxidation states of elements were investigated by X-ray photoelectron spectroscopy (XPS; Thermo Fisher Scientific, Waltham, MA, USA) under high vacuum. All powder samples were attached to carbon tape on a sample holder and dried under a vacuum overnight.

The photoluminescence (PL) emission spectra were analyzed using Shimadzu RF-5301PC; λ_ex_ = 420 nm. The energy-resolved distribution of electron traps (ERDT) patterns and conduction band bottom (CBB) position were investigated by reversed double-beam photoacoustic spectroscopy (RDB-PAS) and photoacoustic spectroscopy (PAS), respectively [53]. For the measurement of bandgap to estimate conduction band bottom (CBB) position by photoacoustic spectroscopy (PAS) measurement, a sample holder filled with sample was set in a laboratory-made PAS cell equipped with a MEMS (micro-electro-mechanical system; MEMS Microphone Breakout, INMP401 (ADMP401); SparkFun, Niwot, CO, USA) microphone module and a glass window attached on the upper side of the PAS cell. The PAS cell was sealed and a light generated from a xenon lamp (M10-RP; Bunkoukeiki, Hachioji, Tokyo, Japan) equipped with a grating monochromator and modulated at 270 Hz by a light chopper was irradiated from the upper side of the PAS cell with wavelength scanning from 450 nm to 350 nm with a 1-nm step. PA signal was detected by a digital lock-in amplifier (NF Corporation, LI5630, Yokohama, Japan) and recorded with reference to that of graphite. The bandgap of a sample was determined by extrapolating the linear part in the shorter wavelength region. For the measurement of energy-resolved distribution of electron traps (ERDTs) by RDB-PAS measurement, nitrogen saturated with methanol vapor was flowed through a sample-loaded PAS cell for 30 min (nitrogen flow: 30 mL min^−1^), then the cell was sealed off with bulbs. The PAS cell was set in an acrylic box and nitrogen was passed through the box to fill with nitrogen. Two light beams combined by a UV quartz combiner light guide (MWS5-1000S-UV3; Moritex, Saitama, Japan) were irradiated from the upper side of the PAS cell. One was a monochromatic continuous light generated from a xenon lamp (M10-RP; Bunkoukeiki, Tokyo, Japan) equipped with a grating monochromator scanned from 650 nm to 350 nm with a 5-nm step to excite electrons from VB to ETs. The other was a 625-nm LED light (Quadica Developments, Luxeon LXHL-ND98; Luxeon, San Jose, CA, USA) modulated at 35 Hz by a digital function generator (DF1906; NF Corporation, Yokohama, Japan) to detect PA signal. The PA signal was detected by a digital lock-in amplifier (LI5630; NF Corporation, Yokohama, Japan) and plotted against photon energy of continuous light. The PA intensity was converted into absolute ET density in the unit of µmol g^−1^ eV^−1^ with factor determined for titanium(IV) oxide (titania) from total ET density measured by photochemical method. At present, the factor for carbon nitride to know the absolute density of ETs in carbon nitrides has not been determined and thereby the density is shown as a relative value. The thus-obtained spectrum was differentiated from the lower energy to higher energy to obtain ERDT pattern. To obtain bar-graph type ERDT pattern, the above-mentioned ERDT was replotted with a 0.05-eV pitch.

### 2.4. Photocatalytic Experiments of H_2_ Evolution over S-Doped g-C_3_N_4_ Photocatalyst

The photocatalytic experiments of H_2_ generation were performed under vis irradiation, using triethanolamine (TEOA) and in situ deposited platinum, as a hole scavenger and a co-catalyst, respectively. In a typical procedure, 60 mg of photocatalyst was suspended in 5 mL of an aqueous suspension of 30 vol% TEOA in a 35-mL Pyrex test tube. Then, an aqueous solution of H_2_PtCl_6_·6H_2_O (1–3.5 wt% Pt in respect to g-C_3_N_4_) was added to the photocatalyst suspension, sonicated for 10 min, and then pre-bubbled with argon for 30 min in the dark to remove oxygen. After that, the test tube was sealed with a rubber septum, the suspension was continuously stirred in a thermostated water bath (temperature ~298 K) and irradiated with vis irradiation (450 W-Xe lamp, λ > 400 nm; water IR filter, cold mirror and cut-off filter Y-42).

To achieve the maximum activity of H_2_ generation, the condition-dependent activity was examined, i.e., the effect of initial suspension pH value, the content of co-catalyst (Pt), photocatalyst dose and TEOA content. The recycling experiments were also performed with three repetitions for the most active sample. During the photocatalytic reaction, the amount of evolved-H_2_ gas was analyzed (every 1 h) using a Shimadzu GC-8A chromatograph (Shimadzu Corporation, Kyoto, Japan), equipped with a thermal conductivity detector (TCD) and Porapak Q column (Agilent Technologies, Santa Clara, CA, USA).

## 3. Results and Discussion

### 3.1. Physicochemical Properties

**Figure 1:**
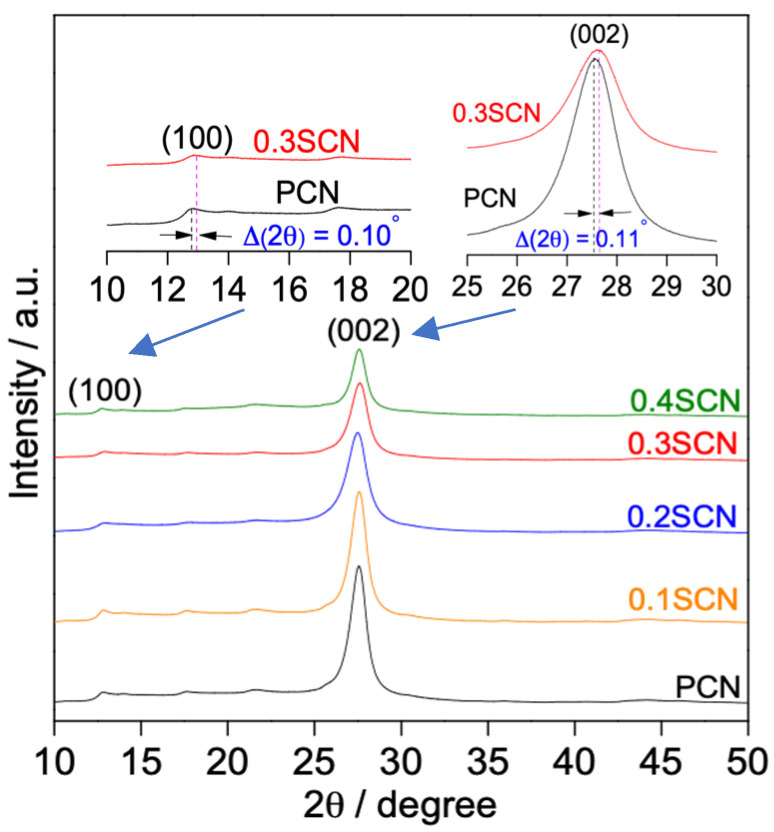
XRD patterns of PCN, 0.1SCN, 0.2SCN, 0.3SCN and 0.4SCN photocatalysts.

The successful preparation of pristine and S-doped g-C_3_N_4_ samples have been confirmed by different methods, including XRD. Indeed, two characteristic XRD peaks of a typical graphitic carbon nitride structure, i.e., (100) and (002) planes at 2θ angles of ca. 13° and 27°, respectively [54,55], are clearly observed in all synthesized samples (Figure 1). The peak at the lower angle (100) is ascribed to the periodic in-plane packing (with a distance of ca. 0.687 nm) of tri-s-triazine motifs in the aromatic systems [56,57], whereas the peak at the higher angle (002) to the periodic interlayer/facial stacking (with a distance of ca. 0.326 nm) of motifs existing in the conjugated aromatic systems, revealing the formation of the pronounced graphitic structure of g-C_3_N_4_ [33,46]. Noticeably, the XRD peaks of both PCN and S-doped CN samples are highly similar, implying a well preserving of the general structure of g-C_3_N_4_ even after S doping [58]. Nevertheless, two distinguished changes have been found after S doping, i.e., the weakening in the intensity and the shifting of 2θ angle to a higher angle value, for the both diffraction peaks. Remarkably, the intensity of the (002) peak of S-doped CN photocatalyst shows a broadening and decreased peak intensity compared with that of PCN, and the intensity of (002) peak decreased with the content of AS. Various reasons of the intensity decrease in XRD peaks, resulting from the modifications of CN framework, have already been proposed, such as lattice distortion, decreasing in the distance of C-N layers, reducing in the planar size of the layers, poor degree in planar structure units and a decrease in crystallinity [25,33,37,46,50,59,60,61,62,63,64]. Therefore, it might be proposed that the nanostructure of g-C_3_N_4_ was shrank to the thinner layers of g-C_3_N_4_ nanosheets by S-doping (but of larger sizes), improving the photocatalytic activity (as shown in the Section 3.2) by possibly shortening the time of electrons’ migration within the layers.

The S doping has been confirmed by obvious shift of both XRD peaks to the higher angles, e.g., for 0.3SCN sample from 12.74° to 12.84° (∆ (2θ) = 0.10°) and from 27.50° to 27.61° (∆ (2θ) = 0.11°) for (100) and (002) planes, respectively (inset Figure 1), indicating a decrease in the inter-planar stacking distance after S-doping. These findings exhibit a good correlation with previous literature [46,65,66]. For example, the (002) diffraction peak of g-C_3_N_4_ shifted from 27.1° to 27.3° and 27.5° after adding ammonium thiosulfate ((NH_4_)_2_S_2_O_3_) and (ammonium thiosulfate + ammonium sulfate ((NH_4_)_2_SO_4_)), respectively (i.e., after S-doping) [46]. It has been proposed that ammonium sulfate might react with CN or its molecular precursors during the thermal-polymerization process, leading to a decrease in ordered structure (i.e., reducing the distance between the C-N layers) in the CN framework by the replacement of carbon (electronegativity = 2.04) with sulfur (electronegativity = 2.19) [33,46].

**Figure 2:**
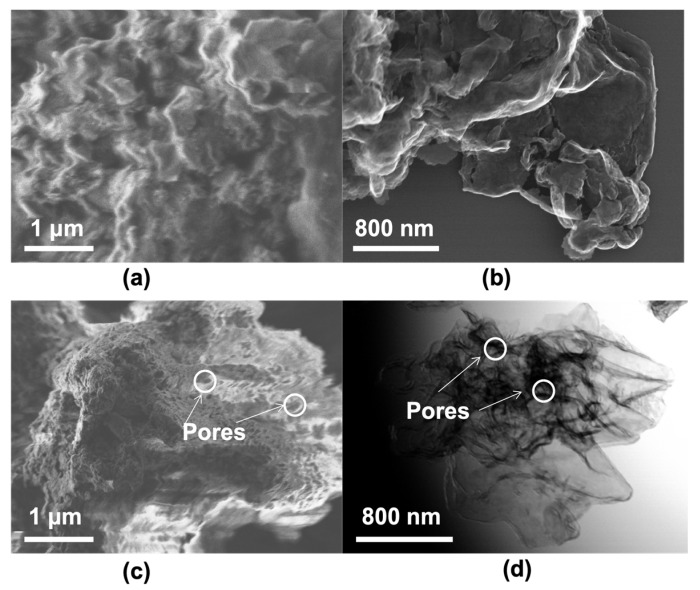
FE-SEM (**a**,**c**) and STEM (**b**,**d**) images of PCN (**a**,**b**) and 0.3SCN (**c**,**d**) photocatalysts.

The layered structure of both pristine and S-doped (0.3SCN) samples have been confirmed by microscopic observations, as exemplary presented in Figure 2. Dense aggregation of many irregular nanosheets during a gas-templating synthesis causes the formation of layered microstructure, as shown in Figure 2a,c. It has already been proposed that a gas-templating method is excellent for the formation of layered structures, because gas bubbles, released by the thermal decomposition of inorganic salts (acting as bubbling or blowing templates; such as ammonium salts) during the thermal-polymerization, could restrict the polymerization of g-C_3_N_4_ into a block, and thus facilitate the formation of ultrathin 2D g-C_3_N_4_ nanosheets with a decentralized perforated layered structure [23,33]. For example, Lu et al. have observed that a release of ammonia gas during synthesis of g-C_3_N_4_ from the mixture of dicyandiamide and ammonium chloride has caused the formation of specific morphology, i.e., ultrathin nanosheets with a crinkly structure [23]. In contrast, the samples prepared in the absence of NH_4_Cl, and thus without ammonia liberation, have been characterized by non-uniform bulk structure.

Interestingly, in the case of pristine g-C_3_N_4_ photocatalyst (PCN), the nanolayers are tightly connected to form a block, i.e., non-porous microstructure (Figure 2a), whereas the S-doped CN (0.3SCN) is porous (Figure 2c), which should result from the release of ammonia gas during the thermal-polymerization of a mixture of urea and ammonium sulfate. It has already been proposed that the porous layered structure of S-doped g-C_3_N_4_ photocatalyst is favorable for the formation of reactive sites on the photocatalyst surface, thus improving the photoinduced charge carriers’ separation and transfer, which is directly connected with boosting the photocatalytic activity (as discussed later) [59]. Furthermore, it is expected that enhanced light reflection and scattering on the layered structure might also result in generation of high number of charge carriers (by reflected/scattered light inside the structure, i.e., efficient light harvesting), improving the overall performance [67]. The layered structure has further been confirmed also by STEM observations (Figure 2b,d). Similar to FE-SEM, STEM images indicate 2D microstructures of “solid” and “nanoporous crumpled lamellar-like” (with several stacking layers nature for PCN and 0.3SCN, respectively.

**Figure 3:**
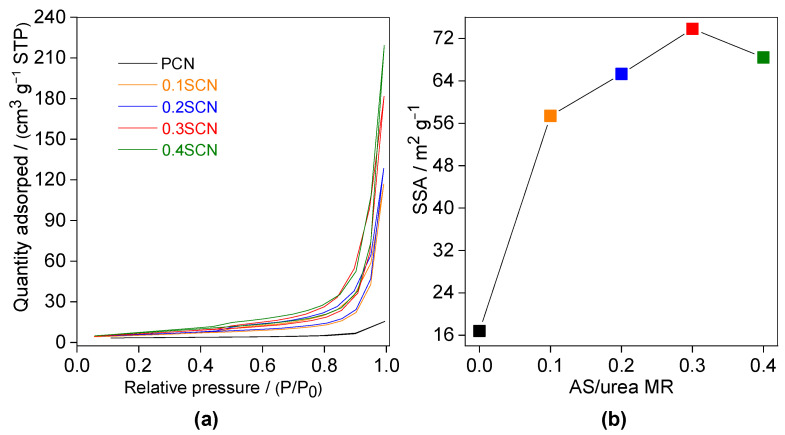
(**a**) N_2_ adsorption-desorption isotherms of PCN, 0.1SCN, 0.2SCN, 0.3SCN and 0.4SCN catalysts; and (**b**) correlation between SSA and AS/urea MR. SSA, specific surface area. AS/urea MR, ammonium sulfate/urea molar ratio.

To confirm the porous nature of S-doped samples, N_2_ adsorption-desorption isotherms for all samples have been studied, and obtained data are shown Figure 3a. Indeed, it has been found that only S-doped samples are porous, with typical type IV isotherms (IUPAC classification) of broad H3 hysteresis loops, indicating the formation of mesoporous CN structure [59]. In contrast, the pristine g-C_3_N_4_ (PCN) photocatalyst shows a typical type III isotherm with a very narrow hysteresis loop (almost disappeared), revealing the formation of CN structure without pores (or with microspores) [33]. Meanwhile, the surface properties of all photocatalysts have been further investigated, and obtained data, e.g., the corresponding pore size (PS) diameter, the pore volume (PV) and specific surface area (SSA), are listed in Table 2. Indeed, the pore size of PCN is 0.75–1.4 nm (micropore), whereas the mesopores appear in the case of S-doped photocatalysts (i.e., 3.9–6.7 nm (0.1SCN), 5.1–7.5 nm (0.2SCN), 5.5–8.1 nm (0.3SCN) and 5.3–7.9 nm (0.4SCN). The pore volume (PV) of PCN, 0.1SCN, 0.2SCN, 0.3SCN and 0.4SCN photocatalysts reaches 0.0014, 0.19, 0.25, 1.3 and 0.98 cm^3^ g^−1^, respectively (Table 2), confirming the block/solid and porous nature of PCN and S-modified CN, respectively. SSA values of all doped samples are much higher (3.4–4.4 times) than that by pristine PCN photocatalyst (16.8 m^2^ g^−1^). Accordingly, the 0.3SCN sample exhibits the largest SSA of 73.8 m^2^ g^−1^, which is ca. 1.1, 1.1, 1.3 and 4.4 times higher than those by 0.4SCN, 0.2SCN, 0.1SCN and PCN (16.8 m^2^ g^−1^) photocatalysts. The SSA values increase with an increase in the amount of AS (AS/urea molar ratio), reaching the optimum for 0.3 molar ratio, and then decreasing (Figure 3b). This might be explained by the fact that all parameters (SSA, PS and PV) increase with an increase in the amount of generated gases during pyrolysis, reaching the optimal value at specific conditions (here, 0.3 molar ratio), whereas the further increase in AS/urea ratio might cause an opposite effect, i.e., decrease in all these values, since too large bubbles could be formed. Similar findings have already been observed by Iqbal and co-workers [68].

**Figure 4:**
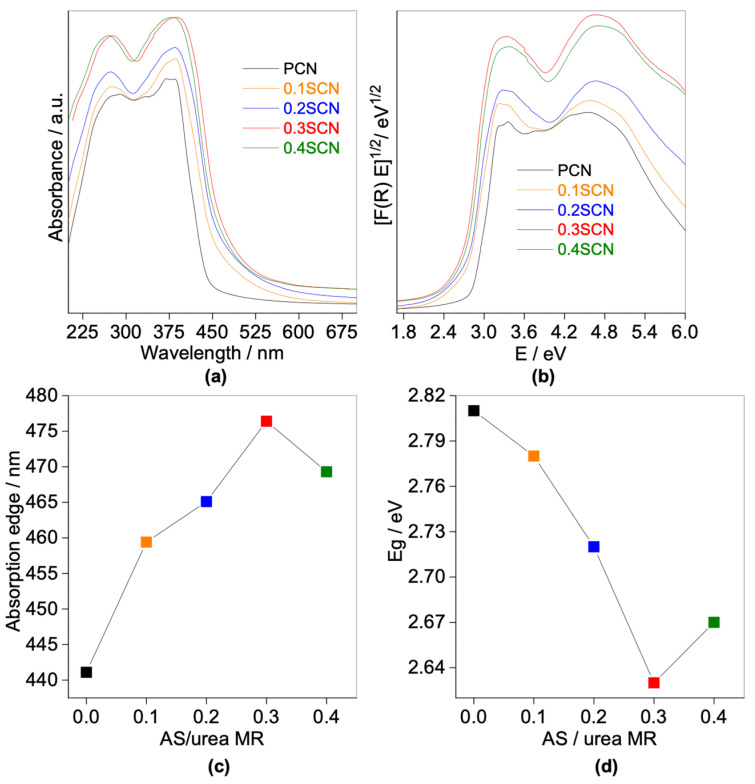
(**a**) UV-vis diffuse reflectance spectra; (**b**) the corresponding Kubelka–Munk transformed reflectance spectra; (**c**,**d**) correlation between AS/urea MR and: (**c**) absorption edge; and (**d**) energy bandgaps.

Photoabsorption properties of obtained samples confirm their response in vis part of solar spectrum with absorption edge (AE) at 441.1–476.4 nm, as shown in Figure 4, and summarized in Table 2. The absorption edge has been shifted bathochromically after S doping, because of band gap narrowing. Accordingly, the pristine photocatalyst (PCN) exhibits the lowest absorption edge and the broadest band gap (441.1 nm and 2.81 eV, respectively), whereas 0.3SCN sample displays the highest absorption edge and the narrowest band gap (i.e., 476.4 nm and 2.63 eV), as shown in Figure 4 and Table 2. The absorption edge/band gap gradually increases/decreases by increasing the content of AS, reaching the maximum/minimum value at 0.3 molar ratio of AS to urea, and then decreases/increases with a further increase in AS content (see Figure 4c,d). Reaching the optimal conditions might be explained by the fact that the sulfur atoms are incorporated into the lattice of g-C_3_N_4_, leading to the formation of new levels between the CB and VB of g-C_3_N_4_, and hence decreasing the energy bandgap of CN. However, the further increase in AS content results in slight blue shift of the absorption edge and broadening of the bandgap, which could be caused by excess liberation of ammonia (during synthesis), and thus insufficient doping rate.

**Figure 5:**
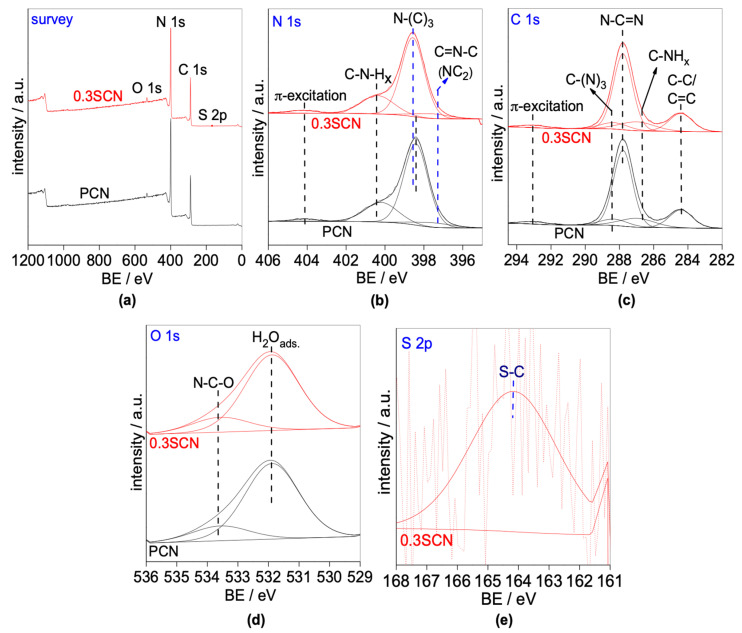
(**a**) XPS survey spectra; and high-resolution XPS spectra: (**b**) N 1s, (**c**) C 1s, (**d**) O 1s of the PCN and 0.3SCN catalysts; and (**e**) high-resolution S 2p spectrum of the 0.3SCN catalyst.

The surface properties and oxidation states of elements have been investigated for PCN and 0.3SCN photocatalysts by XPS analysis, and obtained data are displayed in Figure 5 and Table 3. The wide-scan spectra of PCN and 0.3SCN are shown in Figure 5a. The spectrum of PCN photocatalyst shows mainly three peaks with binding energies of around 399.6, 288.9 and 533.2 eV, characterized for N 1s, C 1s and O 1s elements, respectively, whereas an additional peak at ca. 166.1 eV could be observed for 0.3SCN sample, due to the presence of sulfur (S 2p). As expected, N/C ratio in 0.3SCN sample is lower than that in PCN one and hence anticipates the replacement of the lattice nitrogen by sulfur. In contrast, O/C ratio in 0.3SCN photocatalyst is almost twice as large than that in the pristine sample, suggesting the enrichment of photocatalyst surface with oxygen when doped with sulfur. Therefore, it might be concluded that sulfur is incorporated into the g-C_3_N_4_ lattice with preferential occupation of the nitrogen sites. The narrow-scan spectra of N 1s, C 1s, O 1s and S 2p have also been examined, as shown in Figure 5b–e. In the case of nitrogen, N 1s peaks could be fitted into four peaks at energy of 397.3, 398.4, 400.4 and 404.2 eV. The binding energy of 397.3 eV might be attributed to the pyridinic/triazinic-N (N–(C)_2_; N–C=N; i.e., sp^2^-hybridized N) in the heptazine structure [37,69]. The peak at ca. (398.4) could be ascribed to the pyrrolic-nitrogen (N–(C)_3_; i.e., tertiary nitrogen groups) [37,69], whereas that at 400.4 eV to graphitic-nitrogen (C–NH*_x_*; i.e., terminal amino groups) [37,69]. The binding energy of 404.2 eV could be assigned to π-excitation [70]. Demonstrably, the binding energy of pyrrolic-nitrogen (N–(C)_3_) of doped photocatalyst is slightly shifted (towards larger energy of 398.6 eV), in comparison to the pristine sample, revealing a decrease in the electron density around nitrogen of g-C_3_N_4_ lattice by sulfur doping. Therefore, it might be proposed that some nitrogen atoms in the lattice are replaced by sulfur, and thus the electrons migrate from N to S (of higher electronegativity). In the case of carbon, C 1s peak might also be divided into five parts with binding energies of 284.4, 286.7, 287.8, 288.4 and 293.1 eV, as shown in Figure 5c. The binding energies of 284.4 and 286.7 eV are characteristic for the extraneous carbon (i.e., sp^2^-graphitic carbon; C–C/C=C bonds) and the sp^2^-hybridized carbon (C–NH*_x_* groups; *x* = 1 or 2) on the edges of aromatic units, respectively [37,51,69,71,72]. The binding energies of 287.8, 288.4 and 293.1 eV could be assigned to the sp^2^-bonded carbon (N–C=N; i.e., C-(N)_2_), the C-(N)_3_ groups in the g-C_3_N_4_ framework, and π-excitation, respectively [37,50,51,69,71,72,73,74,75]. The spectra for oxygen (O 1s), displayed in Figure 5d, might fit into two peaks with binding energies of 532.1 and 534.2 eV, characterized to adsorbed-water/hydroxyl anion on the surface of photocatalyst, and C–N–O groups, respectively [76,77]. Furthermore, the wide-scan spectrum of 0.3SCN sample in the S 2p region (164.2 eV) (Figure 5e) indicates S–C bond, formed by the substitution of nitrogen in the g-C_3_N_4_ lattice with sulfur [33,69].

**Figure 6:**
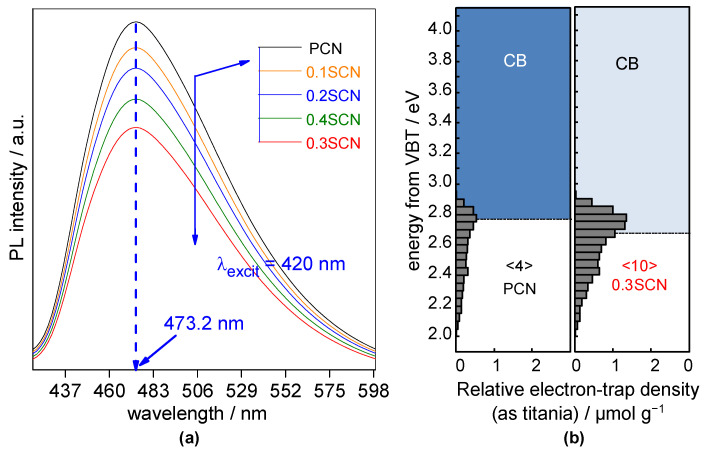
(**a**) PL spectra of the PCN, 0.1SCN, 0.2SCN, 0.3SCN and 0.4SCN photocatalysts; (**b**) Representative energy-resolved distribution of electron traps (ERDTs)/conduction band bottom (CBB) patterns of pristine CN (PCN) and S-doped CN (0.3SCN) photocatalysts. The numbers in brackets (< >) denote the relative total electron-trap density of ETs, with the units of μmol g^−1^. VBT, valence band top.

To investigate the electronic properties of obtained photocatalysts, PL and RDB-PAS spectroscopic analyses have been performed, and obtained data are shown in Figure 6. The photoluminescence is observed in all samples at ca. 473 nm (after excitation at 420 nm), correlating well with the recombination of photoinduced charge carriers (electrons and holes) [2]. It should be pointed out that PL intensity differs significantly between samples. Clearly, the highest intensity, and thus probable the fastest charge carriers’ recombination [59], is observed for the pristine CN (PCN) sample. In contrast, PL intensities of S-doped photocatalysts are weaker, proving the fast charge carriers’ separation, as already reported for other S-doped g-C_3_N_4_ materials [33,46,69,73,74]. Similar to other properties, the optimal synthesis conditions have been confirmed for 0.3SCN sample with the lowest intensity of PL. The decrease in PL intensity with an increase in AS/urea molar ratio till 0.3 value, and the further increase might suggest that too large AS content during synthesis results in the formation of large ammonia bubbles, worsening both physicochemical properties and photocatalytic activity of obtained photocatalysts (as shown above for 0.4SCN sample). It is also possible that the excessive content of sulfur (above the optimal value) might result in the formation of also recombination sites. Nevertheless, it is expected that all S-doped samples, especially 0.3SCN one, should exhibit better photocatalytic performance than pristine one.

Next, RDB-PAS spectroscopic analysis has been performed to measure the energy-resolved distribution of electron traps (ERDTs) patterns with conduction band bottom (CBB) of pristine CN (PCN) and S-doped CN (0.3SCN) photocatalysts, and obtained results are shown in Figure 6b. Two samples show similar levels of accumulated electrons in the ETs around 2.7 eV, which might be a characteristic feature of carbon nitride [78]. The PCN and 0.3SCN samples displayed the ERDT/CBB patterns with the energy range of ~2–2.90 eV, but the electron accumulation density of bare CN is much lower than that of S-doped CN. Chuaicham et al. suggested that pure g-C_3_N_4_ displays single peak at ca. 2.7–2.8 eV and the lower energy tailing up to ca. 2 eV might be assigned to the surface oxidation since the photocatalyst was heated in air, tending to give this low-energy tailing [78]. Moreover, in the case of oxygen and sulfur modification of g-C_3_N_4_, the characteristic peaks at ca. 2.2–2.4 eV were detected in their study [78]. Here, no clear peak could be seen, which could be caused by uniform doping of sulfur inside the structure rather than “doping like” surface modification (no shift of XRD peak in the mentioned study [78]). Additionally, it should be pointed out that the ERDT patterns, conduction band bottom (CBB) and total electron-trap (ET) density might reflect the surface structure, bulk structure and surface/bulk size, respectively [79]. It has been reported that the total density (TD) of ETs is roughly proportional to specific surface area. Therefore, the difference in TD of two samples might reflect the difference in specific surface area, i.e., the larger total ETs density (<ETs>) of the 0.3SCN sample (of ca. 2.5 times than that of PCN) confirms the larger specific surface area of 0.3SCN photocatalyst.

### 3.2. Photocatalytic H_2_ Generation

**Figure 7:**
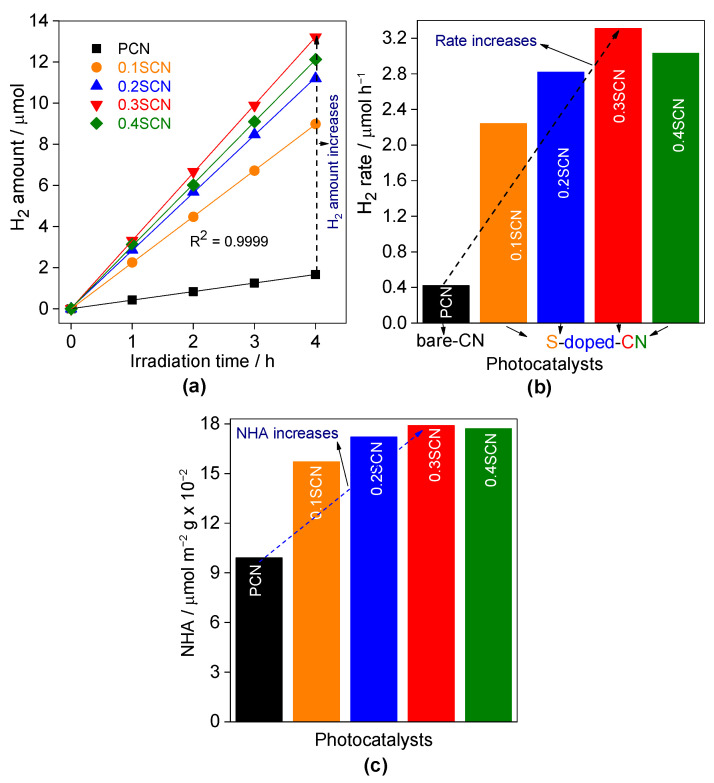
Photocatalytic activity of H_2_ generation on the PCN, 0.1SCN, 0.2SCN, 0.3SCN and 0.4SCN photocatalysts under vis irradiation: (**a**) generated-H_2_ amount; and (**b**) H_2_ evolution rate; (**c**) normalized data of H_2_ evolution rate per specific surface area. Photocatalyst dose: 60 mg; Pt loading: 3 wt%; TEOA vol%: 30; pH value: 6.5; T: 298 K and the reaction volume: 5 mL.

The photocatalytic performance of obtained samples has been examined for hydrogen evolution under vis irradiation in the presence of in situ deposited platinum as a co-catalyst. It has been found that H_2_ is not formed in the absence of light (dark) or photocatalyst (photolysis) in the system. Therefore, it might be concluded that hydrogen evolution proceeds via photocatalytic mechanism. It has been found that a linear increase in the H_2_ amount has been observed for all samples during 4-h vis irradiation, suggesting the high photo-stability of all photocatalysts. As expected, pristine sample (PCN) exhibits the lowest activity (0.42 µmol h^−1^), as shown in Figure 7a,b; and Table 4. Many reasons could be proposed, such as the low SSA (16.8 m^2^ g^−1^), non-porous structure, low visible-light absorption ability and the fast charge carriers’ recombination rate, as already reported [80]. It is clear that the photocatalytic activity of g-C_3_N_4_ is significantly improved by S-doping, especially in the case of the best sample, i.e., 0.3SCN, with activity of ca. eight times larger. Similar to all other properties, the photocatalytic activity increases with an increase in the AS/urea molar ratio from 0.0 to 0.3, and then decreases at 0.4 molar ratio.

**Figure 8:**
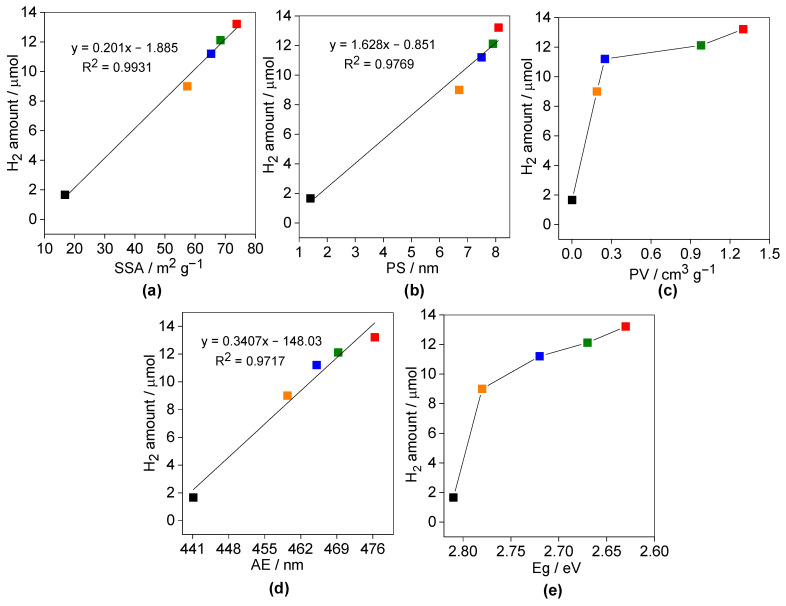
Correlation between the photocatalytic activity (H_2_ generation) and proprties: (**a**) SSA, (**b**) PS, (**c**) PV, (**d**) AE and (**e**) Eg.

It has been found that photocatalytic activity correlates well with all properties, i.e., SSA (Figure 8a), PS (Figure 8b), PV (Figure 8c), AE (Figure 8d) and Eg (Figure 8e). All properties are crucial for the overall photocatalytic performance of the 0.3SCN sample, including high SSA (73.8 m^2^ g^−1^), mesoporous structure (PS = 5.5–8.1 nm) and efficient light harvesting (λ = 476.4 nm; 2.63 eV). However, normalized activity data (e.g., per specific surface area (Figure 7c)) still indicate that there is another factor governing the best photocatalytic performance. Although all modified samples possess similar photoabsorption properties, 0.3SCN is the most active, which suggests that the optimal doping ratio of sulfur could be a key factor for the best performance. It is well known that in the case of doping, the dopants could have double function, i.e., (i) “temporary” charge trapping site, facilitating the charge migration (improved photocatalytic activity) and (ii) “permanent” charge trapping site, resulting in charge carriers’ recombination (decreased photocatalytic activity). Here, besides the nature of dopants, their content is also important to allow uniform doping inside the structure (rather than the formation of some surface modifications). Accordingly, 0.3SCN samples could be considered as that with the optimal doping ratio, which allows the efficient charge migration.

### 3.3. Improvement of Photocatalytic Performance during H_2_ Generation

To achieve the maximum photocatalytic activity during H_2_ evolution on the most active photocatalyst (0.3SCN), the condition-dependent photocatalytic activity has been examined, i.e., the effect of initial pH value, co-catalyst (Pt) loading, photocatalyst dose and sacrificial reagent content (TEOA vol%).

**Figure 9:**
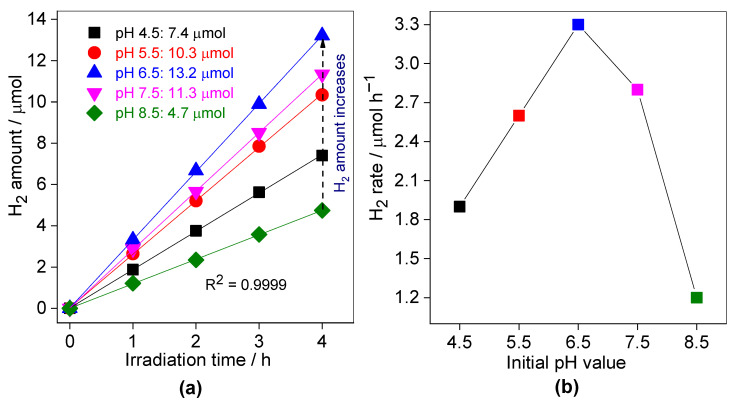
The effect of initial pH value on the photocatalytic activity of H_2_ evolution over the 0.3SCN photocatalyst under vis irradiation: (**a**) generated-H_2_ amount; and (**b**) H_2_ evolution rate. Photocatalyst dose: 60 mg; Pt loading: 3 wt%; TEOA vol%: 30; pH value: 4.5–8.5; T: 298 K and the reaction volume: 5 mL.

The effect of initial pH value of reaction suspension (in the range of 4.5–8.5) on the photocatalytic activity during H_2_ generation in the presence of 60 mg of 0.03SCN photocatalyst in situ loaded with 3 wt% Pt, and in the presence of 30 vol% TEOA has been investigated, and obtained data are shown in Figure 9. It has been found that the photocatalytic activity increases with an increase in the initial pH value from 4.5 to 6.5, and then decreases at pH 8.5. This could be assigned to the charging behaviors of both 0.3SCN photocatalyst surface and TEOA in the solution, altering the electrostatic interaction between the 0.3SCN surface and TEOA [80]. TEOA might be partially ionized and negatively charged at pH 6.5, whereas the 0.3SCN surface might be positively charged, and thus TEOA molecules could adsorb easily on the 0.3SCN surface at pH 6.5, hence improving the photocatalytic performance. In fact, the effect of pH value on photocatalytic evolution of H_2_-fuel is very intricate, and might be assigned to the redox potential ability of the semiconductor in the solution (depending on the positions of the CB and VB of the semiconductor), the charge of the photocatalyst surface with respect to the sacrificial agent charge, diffusion and adsorption of sacrificial agent on the semiconductor surface [81,82].

**Figure 10:**
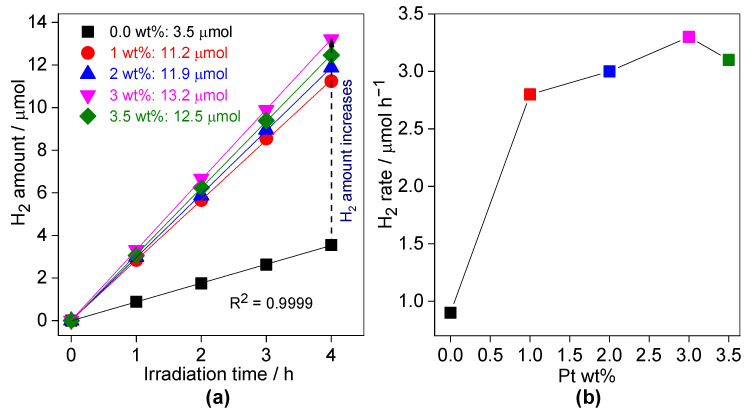
The effect of Pt loading on the photocatalytic activity of H_2_ evolution over the 0.3SCN photocatalyst under vis irradiation: (**a**) generated-H_2_ amount; and (**b**) H_2_ evolution rate. Photocatalyst dose: 60 mg; Pt loading: 0.0–3.5 wt%; TEOA vol%: 30; pH value: 6.5; T: 298 K and the reaction volume: 5 mL.

The effect of Pt loading (in the range of 0.0 to 3.5 wt%) on the photocatalytic activity of H_2_ generation over 60 mg of 0.3SCN photocatalyst at optimal pH value (6.5) and in the presence of 30 vol% TEOA is shown in Figure 10. It is well known that for efficient hydrogen evolution, the presence of a co-catalyst (usually platinum) is necessary due to high over-potential [83]. Indeed, the photocatalytic activity increases significantly after platinum addition. However, it should be pointed out that even without platinum, the reasonable amount of hydrogen has been generated under vis irradiation. The g-C_3_N_4_ photocatalyst might generate the charge carriers under vis irradiation. Then, in the oxygen-free photocatalytic system, the photogenerated holes are consumed by the electron donor and the photoinduced electrons are trapped near the g-C_3_N_4_ surface, probably forming N defect sites in place for proton (^+^H) reduction (in the absence of co-catalyst such as Pt) [84,85]. The Pt co-catalyst on the CN surface might act as a sink (already proven for various other semiconductors [86,87,88]) for the photogenerated electrons, thus improving the electron-hole separation [85,89]. Moreover, it has been proposed that noble-metal co-catalyst works as an active site for the formation of hydrogen molecule, e.g., in the case of Ni/Pd-co modified titania samples [90]. Here, the best performance has been achieved for 3 wt% of Pt loading, i.e., an increase with an increase in its content from 0 to 3 wt%, and then a decrease for 4 wt%. The existence of optimal content of noble metals as a co-catalyst has been commonly reported, mainly from results of the competition for photons between semiconductor and noble metals, i.e., “the shielding effect”, when photons could not reach directly the semiconductor surface [83].

**Figure 11:**
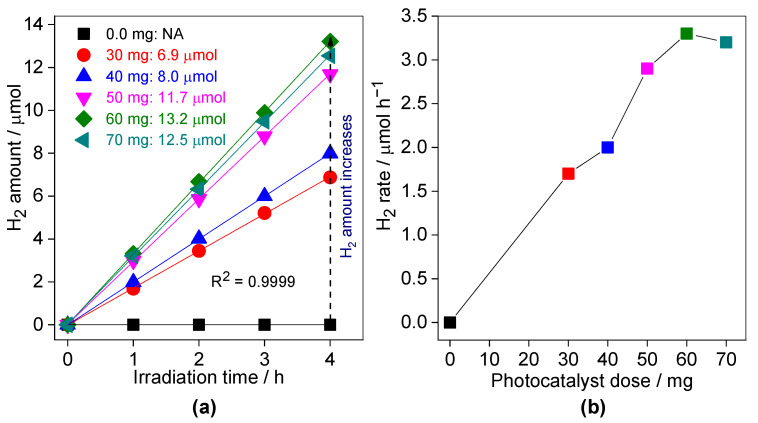
The effect of photocatalyst dose on the photocatalytic activity of H_2_ evolution over the 0.3SCN photocatalyst under vis irradiation: (**a**) generated-H_2_ amount; and (**b**) H_2_ evolution rate. Photocatalyst dose: 0.0–70 mg; Pt loading: 3 wt%; TEOA vol%: 30; pH value: 6.5; T: 298 K and the reaction volume: 5 mL.

Additionally, the effect of photocatalyst (0.3SCN) dose has been investigated in the range of 0.0 to 70 mg, and obtained data are shown in Figure 11. It is well known that photocatalytic activity increases with an increase the content of photocatalyst, reaching a plateau, and then the further increase in its dose might result in a decrease in the overall activity. This aspect has been well discussed by Kisch in pointing out the relevant comparison between different photocatalysts, showing that the activity tests should be performed at plateau regions [91]. Generally, the photocatalytic activity increases with an increase in the photocatalyst content, because of increasing the light absorption, and thus formation of larger number of charge carriers [85,92]. However, the activity usually begins to decline after a plateau region since the light penetration in the reaction suspension starts to reduce, thus the photocatalyst concentration should be optimized [85,92]. Indeed, also in the present study, the maximum efficiency has been observed at optimal dose of photocatalyst of 60 mg.

**Figure 12:**
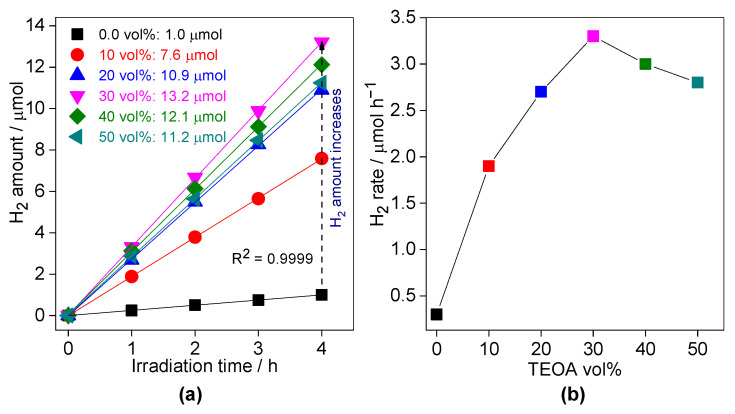
The effect of TEOA vol% on the photocatalytic activity of H_2_ evolution over the 0.3SCN photocatalyst under vis irradiation: (**a**) generated-H_2_ amount; and (**b**) H_2_ evolution rate. Photocatalyst dose: 60 mg; Pt loading: 3 wt%; TEOA vol%: 0–50; pH value: 6.5; T: 298 K and the reaction volume: 5 mL.

Additionally, the dependence of photocatalytic hydrogen evolution on the content of hole scavenger (volume ratio of TEOA to H_2_O) has also been examined. Similar to all other dependences, the optimal value has been notices at 30 vol%, as shown in Figure 12. These results could be explained by the fact that the amount of TEOA adsorbed on the CN photocatalyst surface increases with an increase in its concentration in the suspension, and thus improving the scavenging of photogenerated holes. However, an excessive adsorption of TEAO might also hinder the formation of platinum co-catalyst, and thus the recombination between holes and electrons (not scavenged by platinum) should result in an activity decrease.

Summarizing, the photocatalytic activity of H_2_-fuel generation is strongly dependent on the reaction conditions, such as initial suspension pH value, co-catalyst (Pt) loading, photocatalyst (0.3SCN) dose and hole scavenger (TEOA) content, reaching the best performance for the optimized parameters. Here, the best photocatalytic performance (3.3 µmol h^−1^) under vis irradiation has been obtained for 60 mg of 0.3SCN photocatalyst, with in situ loaded 3 wt% of platinum, in the presence of 30 vol% TEOA and at the initial pH value of 6.5.

### 3.4. Photocatalytic Stability and Reaction Mechanism

**Figure 13:**
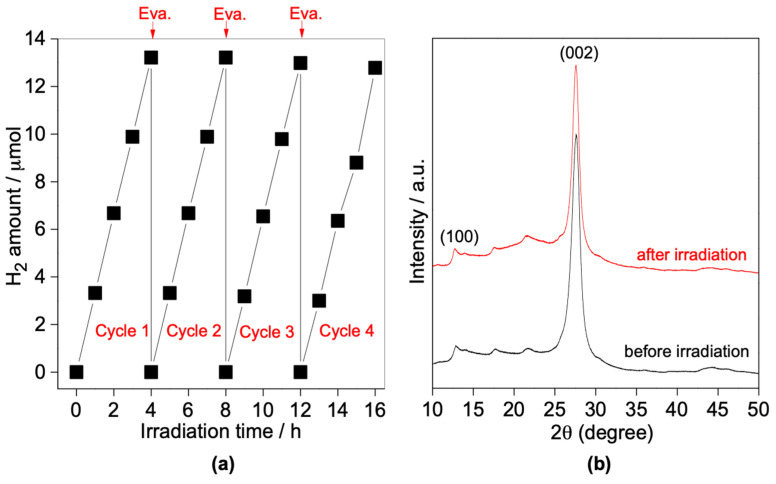
(**a**) Recyclability of the 0.3SCN photocatalyst in the photocatalytic H_2_ generation under vis irradiation. Photocatalyst dose: 60 mg; Pt loading: 3 wt%; TEOA vol%: 30; pH value: 6.5; T: 298 K and the reaction volume: 5 mL. (**b**) XRD patterns of 0.3SCN photocatalyst before and after photocatalytic reaction. (Eva.: evacuation).

The photostability of the as-synthesized S-doped g-C_3_N_4_ photocatalyst, i.e., 0.3SCN sample was investigated by recycling experiments (four times) under vis irradiation. Figure 13a shows the evolved-hydrogen amount over 0.3SCN photocatalyst under vis irradiation during four sequential cycles. It was found that amount of generated H_2_ during the 1st, 2nd, 3rd and 4th cycle reached 13.2, 13.2, 13.0 and 12.8 µmol, respectively, which indicates that there is no an obvious decrease (<1%) in the photocatalytic activity of 2D mesoporous sulfur doped-g-C_3_N_4_ photocatalyst, and thus it might be repeatedly used and recycled. Moreover, the phase structure of 0.3SCN photocatalyst did not change after using it in the photocatalytic reaction (Figure 13b), confirming the high stability of the as-synthesized 0.3SCN photocatalyst.

**Figure 14:**
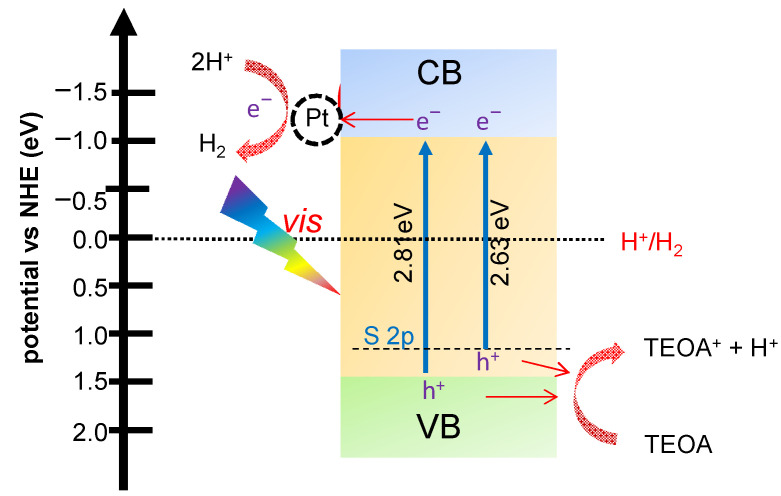
Schematic diagram of the proposed mechanism of photocatalytic H_2_ generation over S-doped CN (0.3SCN) photocatalyst.

Next, the mechanism of the photocatalytic reactions over the as-prepared S-doped g-C_3_N_4_ (0.3SCN) photocatalyst is discussed, as presented in Figure 14. In order to clarify the photoreaction mechanism of sulfur-doped g-C_3_N_4_, the potentials of CB and VB were calculated using the following equations [93]:

E_VB_ = E_CB_ + Eg (1)

E_CB_ = X − E^e^ − 0.5 × Eg (2)

where, E_VB_: potential of VB; E_CB_: potential of CB; Eg: band gap of 0.3SCN (2.63 eV); X: absolute electronegativity of g-C_3_N_4_ (4.73 eV); E^e^: constant relative to the standard hydrogen electrode (4.5 eV). It was found that the calculated E_CB_ and E_VB_ were −1.1 and 1.5 eV, respectively.

The potential of CB minimum (CBM; −1.1 eV) is more negative than the standard redox potential of H^+^/H_2_ (0.0 eV), thus the photogenerated electrons could reduce the H^+^, forming hydrogen molecule on the surface of 0.3SCN. Although hydrogen could be also formed in the absence of platinum (co-catalyst), its presence improves the photocatalytic performance, acting as a sink for photogenerated electrons to avoid both their recombination with photogenerated holes and the formation of some defects on the photocatalyst surface (e.g., N defect sites, self-doping, etc.) (Figure 14). In brief, the electron/hole pairs are generated at the CB/VB of semiconductor (Equation (3)). Then, the photogenerated holes (at the VB) transfer to the photocatalyst surface to oxidize TEOA, forming proton H^+^ (Equation (4)), whereas the photogenerated electrons (at the CB) are trapped onto Pt to reduce the proton into H_2_ gas (Equation (5)). Of course, in the case of S-doping, the narrowing of bandgap results in more efficient light harvesting, and thus improved performance, but does not change the overall mechanism pathway.

PCN/S-doped g-C_3_N_4_ + vis → e^−^ (CB) + h^+^ (VB) (3)

h^+^ (CB) + TEOA → TEOA^+^ + H^+^ (4)

2e^−^ (CB) + 2H^+^ → H_2_ (5)

## 4. Conclusions

The 2D porous S-doped g-C_3_N_4_ photocatalyst could be successfully synthesized by a facile gas-templating method through a simultaneous pyrolysis of urea (main raw material) and (NH_4_)_2_SO_4_ (S source and structure agent). It was demonstrated that ammonium sulfate played the main role to realize simultaneous fabrication of nanostructured morphology and sulfur doping in one-step method to address the limitations of pristine g-C_3_N_4_ (i.e., fast charge carriers’ recombination, small surface area and low visible-light Tabsorption ability). The as-prepared photocatalysts, characterized by diverse analytic methods (i.e., XRD, FE-SEM, STEM, BET-surface area, UV-vis-DRS, XPS, PL and RDB-PAS), prove to be an efficient material for hydrogen generation under vis irradiation. This reflects the significant role of (NH_4_)_2_SO_4_ to improve the activity of g-C_3_N_4_ by: (i) the formation of 2D and porous morphology (higher specific surface area and good electrical conductivity), (ii) extension of the visible-light absorption (S-doping) and (iii) improvement of the charge carriers’ separation and transfer (temporary charge trapping). Moreover, it has been found that by the optimization of reaction parameters, i.e., initial pH value, co-catalyst (Pt) loading, photocatalyst dose and sacrificial reagent volume ratio (TEOA vol%), high rate of “solar” hydrogen could be formed. Although all reactions parameters seem to be important for the overall performance, it is thought that efficient doping of sulfur is a key factor of photocatalytic activity. This work provides a broadening window for the development of doped g-C_3_N_4_ with outstanding activity for large-scale applications.

## Figures and Tables

**Table 1:** S-doped g-C_3_N_4_ (CN) photocatalysts and their applications.

Materials		Light Source	Application	Activity	Ref.
Raw Material	Sulfur Source				
thiourea		300-W Xe lamp, λ > 400 nm; cut-off filter Y-42	H_2_ generation	5.3 × higher activity of doped CN	[25]
dicyandiamide	H_2_S	300-W Xe lamp, λ > 400 nm; cut-off filter Y-42	H_2_ generation	8 × higher activity of doped CN	[47]
melamine	thiourea	300-W Xe lamp	CO_2_ reduction	1.4 × higher activity of doped CN	[48]
thiourea		300-W halogen lamp; λ > 400 nm	methyl orange degradation	9.4 × higher activity of doped CN	[49]
thiourea		500-W Xe lamp	N_2_ fixation	2.8 × higher activity of doped CN	[50]
dicyandiamide	trithiocyanuric acid	300-W Xe lamp	tetracycline degradation	20 × higher activity of doped CN	[51]
urea	ammonium sulfate	450-W Xe lamp, λ > 400 nm; water IR filter, cold mirror and cut-off filter (Y-42)	H_2_ generation	8 × higher activity of doped CN	This work

**Table 2:** Preparation condition, textural and optical properties of the PCN, 0.1SCN, 0.2SCN, 0.3SCN and 0.4SCN catalysts.

Catalyst Code	AS/Urea Molar Ratio	SSA/ m^2^ g^−1^	PS/ nm	PV/ cm^3^ g^−1^	AE/ nm	Eg/ eV
PCN	0.0	16.8	0.75–1.4	0.0014	441.1	2.81
0.1SCN	0.1	57.4	3.9–6.7	0.19	459.4	2.78
0.2SCN	0.2	65.3	5.1–7.5	0.25	465.1	2.72
0.3SCN	0.3	73.8	5.5–8.1	1.3	476.4	2.63
0.4SCN	0.4	68.4	5.3–7.9	0.98	469.3	2.67

AE: absorption edge; AS: ammonium sulfate; Eg: energy bandgap; SSA: specific surface area; PS: pore size; PV: pore volume.

**Table 3:** Elemental composition and core-levels’ binding energy of N 1s, C 1s, O 1s and S 2p, obtained from the XPS analysis of the PCN and 0.3SCN catalysts.

XPS Data			Photocatalyst	
			PCN	0.3SCN
Elemental composition	atomic %	N 1s	55.40	53.10
		C 1s	42.60	43.28
		O 1s	2.00	3.21
		S 2p	-	0.41
	atomic ratio	N/C	1.30	0.047
		O/C	1.23	0.074
Core-levels (BE/eV)	N 1s	Pyridinic-N: N–(C)_2_; N–C=N	397.3	397.3
		Pyrrolic-N: N–(C)_3_	398.4	398.6
		Graphitic-N: C–NH*_x_*	400.4	400.4
		π-excitation	404.2	404.2
	C 1s	C–C/C=C	284.4	284.4
		C–NH*_x_*	286.7	286.7
		N–C=N	287.8	287.8
		C–(N)_3_	288.4	288.4
		π-excitation	293.1	293.1
	O 1s	O–H/adsorbed water	532.1	532.1
		C–N–O	534.2	534.2
	S 2p	S–C	-	164.2

**Table 4:** The photocatalytic activities of H_2_ generation over the as-prepared CN photocatalysts under vis irradiation.

Photocatalyst Code	H_2_ Amount/μmol	H_2_ Rate/μmol h^−1^
PCN	1.66	0.42
0.1SCN	9.00	2.24
0.2SCN	11.20	2.82
0.3SCN	13.21	3.31
0.4SCN	12.12	3.03

## Data Availability

The data presented in this study are available on request from corresponding author (T.M.K.).
